# Supplementary material for: Smooth Interpolating Curves with Local Control and Monotone Alternating Curvature
Source: Comput Graph Forum. 2022 Oct 6;41(5):25–38. doi: 10.1111/cgf.14600 (PMC9827861; doi:10.1111/cgf.14600)
Supplement: Supplementary file 1 — Supplement Material [file CGF-41-25-s001.zip › Local-Smooth-Interpolating-MonoCurvature/extern/clothoids/docs/genindex.html]

Index — Clothoids v2.0.9

### Navigation

- index
- toc
- Clothoids »
- Index

# Index

**A**
| **B**
| **C**
| **F**
| **G**
| **I**
| **L**
| **M**
| **N**
| **O**
| **P**
| **R**
| **S**
| **T**
| **X**
| **Y**

## A

|  |  |
| --- | --- |
| - AABBtree (class) - AABBtree::AABBtree (function) - AABBtree::bbox (function) - AABBtree::build (function) - AABBtree::clear (function) - AABBtree::collision (function) - AABBtree::empty (function) - AABBtree::intersect (function) - AABBtree::min\_distance (function) - AABBtree::PairPtrBBox (C++ type) - AABBtree::print (function) - AABBtree::PtrAABB (C++ type) - AABBtree::PtrBBox (C++ type) - AABBtree::VecPairPtrBBox (C++ type) - AABBtree::VecPtrBBox (C++ type) | - AABBtree::~AABBtree (function) - AsyPlot (class) - AsyPlot::AsyPlot (function) - AsyPlot::displayAxes (function) - AsyPlot::dot (function) - AsyPlot::drawClothoid (function) - AsyPlot::drawLine (function) - AsyPlot::drawRect (function) - AsyPlot::label (function) - AsyPlot::triangle (function) - AsyPlot::~AsyPlot (function) - Atanc (function) - Atanc\_D (function) - Atanc\_DD (function) - Atanc\_DDD (function) |

## B

|  |  |
| --- | --- |
| - BaseCurve (class) - BaseCurve::BaseCurve (function) - BaseCurve::bbox (function) - BaseCurve::bbox\_ISO (function) - BaseCurve::bbox\_SAE (function) - BaseCurve::bbTriangles (function) - BaseCurve::bbTriangles\_ISO (function) - BaseCurve::bbTriangles\_SAE (function) - BaseCurve::changeOrigin (function) - BaseCurve::closestPoint\_ISO (function), [1] - BaseCurve::closestPoint\_SAE (function), [1] - BaseCurve::collision (function) - BaseCurve::collision\_ISO (function) - BaseCurve::collision\_SAE (function) - BaseCurve::distance (function) - BaseCurve::distance\_ISO (function) - BaseCurve::distance\_SAE (function) - BaseCurve::eval (function) - BaseCurve::eval\_D (function) - BaseCurve::eval\_DD (function) - BaseCurve::eval\_DDD (function) - BaseCurve::eval\_ISO (function) - BaseCurve::eval\_ISO\_D (function) - BaseCurve::eval\_ISO\_DD (function) - BaseCurve::eval\_ISO\_DDD (function) - BaseCurve::eval\_SAE (function) - BaseCurve::eval\_SAE\_D (function) - BaseCurve::eval\_SAE\_DD (function) - BaseCurve::eval\_SAE\_DDD (function) - BaseCurve::evaluate (function) - BaseCurve::evaluate\_ISO (function) - BaseCurve::evaluate\_SAE (function) - BaseCurve::findST\_ISO (function) - BaseCurve::findST\_SAE (function) - BaseCurve::info (function) - BaseCurve::intersect (function) - BaseCurve::intersect\_ISO (function) - BaseCurve::intersect\_SAE (function) - BaseCurve::kappa (function) - BaseCurve::kappa\_D (function) - BaseCurve::kappa\_DD (function) - BaseCurve::kappaBegin (function) - BaseCurve::kappaEnd (function) - BaseCurve::length (function) - BaseCurve::length\_ISO (function) - BaseCurve::length\_SAE (function) - BaseCurve::nor\_ISO (function) - BaseCurve::nor\_ISO\_D (function) - BaseCurve::nor\_ISO\_DD (function) - BaseCurve::nor\_ISO\_DDD (function) - BaseCurve::nor\_SAE (function) - BaseCurve::nor\_SAE\_D (function) - BaseCurve::nor\_SAE\_DD (function) - BaseCurve::nor\_SAE\_DDD (function) - BaseCurve::nx\_Begin\_ISO (function) - BaseCurve::nx\_Begin\_SAE (function) - BaseCurve::nx\_End\_ISO (function) - BaseCurve::nx\_End\_SAE (function) - BaseCurve::nx\_ISO (function) - BaseCurve::nx\_ISO\_D (function) - BaseCurve::nx\_ISO\_DD (function) - BaseCurve::nx\_ISO\_DDD (function) - BaseCurve::nx\_SAE (function) - BaseCurve::nx\_SAE\_D (function) - BaseCurve::nx\_SAE\_DD (function) - BaseCurve::nx\_SAE\_DDD (function) - BaseCurve::ny\_Begin\_ISO (function) - BaseCurve::ny\_Begin\_SAE (function) - BaseCurve::ny\_End\_ISO (function) - BaseCurve::ny\_End\_SAE (function) - BaseCurve::ny\_ISO (function) - BaseCurve::ny\_ISO\_D (function) - BaseCurve::ny\_ISO\_DD (function) - BaseCurve::ny\_ISO\_DDD (function) - BaseCurve::ny\_SAE (function) - BaseCurve::ny\_SAE\_D (function) - BaseCurve::ny\_SAE\_DD (function) - BaseCurve::ny\_SAE\_DDD (function) - BaseCurve::reverse (function) - BaseCurve::rotate (function) - BaseCurve::scale (function) - BaseCurve::tg (function) - BaseCurve::tg\_D (function) - BaseCurve::tg\_DD (function) - BaseCurve::tg\_DDD (function) - BaseCurve::theta (function) - BaseCurve::theta\_D (function) - BaseCurve::theta\_DD (function) - BaseCurve::theta\_DDD (function) - BaseCurve::thetaBegin (function) - BaseCurve::thetaEnd (function) - BaseCurve::translate (function) - BaseCurve::trim (function) - BaseCurve::tx (function) - BaseCurve::tx\_Begin (function) - BaseCurve::tx\_D (function) - BaseCurve::tx\_DD (function) - BaseCurve::tx\_DDD (function) - BaseCurve::tx\_End (function) - BaseCurve::ty (function) - BaseCurve::ty\_Begin (function) - BaseCurve::ty\_D (function) - BaseCurve::ty\_DD (function) - BaseCurve::ty\_DDD (function) - BaseCurve::ty\_End (function) - BaseCurve::type (function) - BaseCurve::X (function) - BaseCurve::X\_D (function) - BaseCurve::X\_DD (function) - BaseCurve::X\_DDD (function) - BaseCurve::X\_ISO (function) - BaseCurve::X\_ISO\_D (function) - BaseCurve::X\_ISO\_DD (function) - BaseCurve::X\_ISO\_DDD (function) - BaseCurve::X\_SAE (function) - BaseCurve::X\_SAE\_D (function) - BaseCurve::X\_SAE\_DD (function) - BaseCurve::X\_SAE\_DDD (function) - BaseCurve::xBegin (function) - BaseCurve::xBegin\_ISO (function) - BaseCurve::xBegin\_SAE (function) - BaseCurve::xEnd (function) - BaseCurve::xEnd\_ISO (function) - BaseCurve::xEnd\_SAE (function) - BaseCurve::Y (function) - BaseCurve::Y\_D (function) - BaseCurve::Y\_DD (function) - BaseCurve::Y\_DDD (function) - BaseCurve::Y\_ISO (function) - BaseCurve::Y\_ISO\_D (function) - BaseCurve::Y\_ISO\_DD (function) - BaseCurve::Y\_ISO\_DDD (function) - BaseCurve::Y\_SAE (function) - BaseCurve::Y\_SAE\_D (function) - BaseCurve::Y\_SAE\_DD (function) - BaseCurve::Y\_SAE\_DDD (function) - BaseCurve::yBegin (function) - BaseCurve::yBegin\_ISO (function) - BaseCurve::yBegin\_SAE (function) - BaseCurve::yEnd (function) - BaseCurve::yEnd\_ISO (function) - BaseCurve::yEnd\_SAE (function) - BaseCurve::~BaseCurve (function) - BBox (class) - BBox::BBox (function), [1] - BBox::collision (function) - BBox::distance (function) - BBox::Id (function) - BBox::Ipos (function) - BBox::join (function) - BBox::maxDistance (function) - BBox::operator= (function) - BBox::print (function) - BBox::PtrBBox (C++ type) - BBox::Xmax (function) - BBox::Xmin (function) - BBox::Ymax (function) - BBox::Ymin (function) - Biarc (class), [1] - Biarc::bbox (function), [1] - Biarc::bbox\_ISO (function) - Biarc::bbox\_SAE (function) - Biarc::bbTriangles (function), [1] - Biarc::bbTriangles\_ISO (function) - Biarc::bbTriangles\_SAE (function) - Biarc::Biarc (function), [1], [2], [3], [4] - Biarc::build (function), [1] - Biarc::build\_3P (function), [1] - Biarc::C0 (function) - Biarc::C1 (function) - Biarc::changeOrigin (function), [1] - Biarc::closestPoint (function) - Biarc::closestPoint\_ISO (function), [1] - Biarc::closestPoint\_SAE (function), [1] - Biarc::collision (function), [1], [2] - Biarc::collision\_ISO (function), [1] - Biarc::collision\_SAE (function) - Biarc::copy (function), [1] - Biarc::delta\_theta (function) - Biarc::distance (function), [1] - Biarc::distance\_ISO (function) - Biarc::distance\_SAE (function) - Biarc::eval (function), [1] - Biarc::eval\_D (function), [1] - Biarc::eval\_DD (function), [1] - Biarc::eval\_DDD (function), [1] - Biarc::eval\_ISO (function) - Biarc::eval\_ISO\_D (function) - Biarc::eval\_ISO\_DD (function) - Biarc::eval\_ISO\_DDD (function) - Biarc::eval\_SAE (function) - Biarc::eval\_SAE\_D (function) - Biarc::eval\_SAE\_DD (function) - Biarc::eval\_SAE\_DDD (function) - Biarc::evaluate (function), [1] - Biarc::evaluate\_ISO (function) - Biarc::evaluate\_SAE (function) - Biarc::find\_coord (function) - Biarc::findST\_ISO (function) - Biarc::findST\_SAE (function) - Biarc::getCircles (function) - Biarc::getData (function) - Biarc::info (function), [1] - Biarc::intersect (function), [1], [2] - Biarc::intersect\_ISO (function), [1] - Biarc::intersect\_SAE (function) - Biarc::is\_type (function) - Biarc::kappa (function), [1] - Biarc::kappa0 (function), [1] - Biarc::kappa1 (function), [1] - Biarc::kappa\_D (function), [1] - Biarc::kappa\_DD (function), [1] - Biarc::kappaBegin (function), [1] - Biarc::kappaEnd (function), [1] - Biarc::length (function), [1] - Biarc::length0 (function), [1] - Biarc::length1 (function), [1] - Biarc::length\_ISO (function) - Biarc::length\_SAE (function) - Biarc::noAABBtree (function) - Biarc::nor\_ISO (function) - Biarc::nor\_ISO\_D (function) - Biarc::nor\_ISO\_DD (function) - Biarc::nor\_ISO\_DDD (function) - Biarc::nor\_SAE (function) - Biarc::nor\_SAE\_D (function) - Biarc::nor\_SAE\_DD (function) - Biarc::nor\_SAE\_DDD (function) - Biarc::nx\_Begin\_ISO (function) - Biarc::nx\_Begin\_SAE (function) - Biarc::nx\_End\_ISO (function) - Biarc::nx\_End\_SAE (function) - Biarc::nx\_ISO (function) - Biarc::nx\_ISO\_D (function) - Biarc::nx\_ISO\_DD (function) - Biarc::nx\_ISO\_DDD (function) - Biarc::nx\_SAE (function) - Biarc::nx\_SAE\_D (function) - Biarc::nx\_SAE\_DD (function) - Biarc::nx\_SAE\_DDD (function) - Biarc::ny\_Begin\_ISO (function) - Biarc::ny\_Begin\_SAE (function) - Biarc::ny\_End\_ISO (function) - Biarc::ny\_End\_SAE (function) - Biarc::ny\_ISO (function) - Biarc::ny\_ISO\_D (function) - Biarc::ny\_ISO\_DD (function) - Biarc::ny\_ISO\_DDD (function) - Biarc::ny\_SAE (function) - Biarc::ny\_SAE\_D (function) - Biarc::ny\_SAE\_DD (function) - Biarc::ny\_SAE\_DDD (function) - Biarc::obj\_handle (function) - Biarc::operator<< (function) - Biarc::operator= (function) - Biarc::plot (function) - Biarc::plotAngle (function) - Biarc::plotBBox (function) | - Biarc::plotCurvature (function) - Biarc::plotNormal (function) - Biarc::plotTBox (function) - Biarc::plotTriangles (function) - Biarc::points (function) - Biarc::reverse (function), [1] - Biarc::rotate (function), [1] - Biarc::scale (function), [1] - Biarc::tg (function) - Biarc::tg\_D (function) - Biarc::tg\_DD (function) - Biarc::tg\_DDD (function) - Biarc::theta (function), [1] - Biarc::theta\_D (function), [1] - Biarc::theta\_DD (function), [1] - Biarc::theta\_DDD (function), [1] - Biarc::thetaBegin (function), [1] - Biarc::thetaEnd (function), [1] - Biarc::thetaMiddle (function), [1] - Biarc::to\_nurbs (function) - Biarc::translate (function), [1] - Biarc::trim (function), [1] - Biarc::tx (function) - Biarc::tx\_Begin (function) - Biarc::tx\_D (function) - Biarc::tx\_DD (function) - Biarc::tx\_DDD (function) - Biarc::tx\_End (function) - Biarc::ty (function) - Biarc::ty\_Begin (function) - Biarc::ty\_D (function) - Biarc::ty\_DD (function) - Biarc::ty\_DDD (function) - Biarc::ty\_End (function) - Biarc::type (function) - Biarc::X (function) - Biarc::X\_D (function) - Biarc::X\_DD (function) - Biarc::X\_DDD (function) - Biarc::X\_ISO (function) - Biarc::X\_ISO\_D (function) - Biarc::X\_ISO\_DD (function) - Biarc::X\_ISO\_DDD (function) - Biarc::X\_SAE (function) - Biarc::X\_SAE\_D (function) - Biarc::X\_SAE\_DD (function) - Biarc::X\_SAE\_DDD (function) - Biarc::xBegin (function), [1] - Biarc::xBegin\_ISO (function) - Biarc::xBegin\_SAE (function) - Biarc::xEnd (function), [1] - Biarc::xEnd\_ISO (function) - Biarc::xEnd\_SAE (function) - Biarc::xMiddle (function), [1] - Biarc::xyBegin (function) - Biarc::xyEnd (function) - Biarc::Y (function) - Biarc::Y\_D (function) - Biarc::Y\_DD (function) - Biarc::Y\_DDD (function) - Biarc::Y\_ISO (function) - Biarc::Y\_ISO\_D (function) - Biarc::Y\_ISO\_DD (function) - Biarc::Y\_ISO\_DDD (function) - Biarc::Y\_SAE (function) - Biarc::Y\_SAE\_D (function) - Biarc::Y\_SAE\_DD (function) - Biarc::Y\_SAE\_DDD (function) - Biarc::yBegin (function), [1] - Biarc::yBegin\_ISO (function) - Biarc::yBegin\_SAE (function) - Biarc::yEnd (function), [1] - Biarc::yEnd\_ISO (function) - Biarc::yEnd\_SAE (function) - Biarc::yesAABBtree (function) - Biarc::yMiddle (function), [1] - Biarc::~Biarc (function) - BiarcList (class), [1] - BiarcList::append (function) - BiarcList::bbox (function), [1] - BiarcList::bbox\_ISO (function) - BiarcList::bbox\_SAE (function) - BiarcList::bbTriangles (function), [1] - BiarcList::bbTriangles\_ISO (function) - BiarcList::bbTriangles\_SAE (function) - BiarcList::BiarcList (function), [1], [2], [3], [4], [5], [6], [7] - BiarcList::build\_G1 (function), [1], [2] - BiarcList::build\_theta (function) - BiarcList::changeOrigin (function), [1] - BiarcList::closestPoint (function) - BiarcList::closestPoint\_ISO (function), [1] - BiarcList::closestPoint\_SAE (function), [1] - BiarcList::collision (function), [1], [2] - BiarcList::collision\_ISO (function), [1] - BiarcList::collision\_SAE (function) - BiarcList::copy (function), [1] - BiarcList::distance (function), [1] - BiarcList::distance\_ISO (function) - BiarcList::distance\_SAE (function) - BiarcList::eval (function), [1] - BiarcList::eval\_D (function), [1] - BiarcList::eval\_DD (function), [1] - BiarcList::eval\_DDD (function), [1] - BiarcList::eval\_ISO (function) - BiarcList::eval\_ISO\_D (function) - BiarcList::eval\_ISO\_DD (function) - BiarcList::eval\_ISO\_DDD (function) - BiarcList::eval\_SAE (function) - BiarcList::eval\_SAE\_D (function) - BiarcList::eval\_SAE\_DD (function) - BiarcList::eval\_SAE\_DDD (function) - BiarcList::evaluate (function), [1] - BiarcList::evaluate\_ISO (function) - BiarcList::evaluate\_SAE (function) - BiarcList::find\_coord (function) - BiarcList::find\_coord1 (function) - BiarcList::findAtS (function) - BiarcList::findST1 (function), [1] - BiarcList::findST\_ISO (function) - BiarcList::findST\_SAE (function) - BiarcList::get (function), [1] - BiarcList::getAtS (function) - BiarcList::getSTK (function) - BiarcList::getXY (function), [1] - BiarcList::info (function), [1] - BiarcList::init (function) - BiarcList::intersect (function), [1], [2] - BiarcList::intersect\_ISO (function), [1] - BiarcList::intersect\_SAE (function) - BiarcList::is\_type (function) - BiarcList::kappa (function), [1] - BiarcList::kappa\_D (function), [1] - BiarcList::kappa\_DD (function), [1] - BiarcList::kappaBegin (function), [1] - BiarcList::kappaEnd (function), [1] - BiarcList::length (function), [1] - BiarcList::length\_ISO (function) - BiarcList::length\_SAE (function) - BiarcList::noAABBtree (function) - BiarcList::nor\_ISO (function) - BiarcList::nor\_ISO\_D (function) - BiarcList::nor\_ISO\_DD (function) - BiarcList::nor\_ISO\_DDD (function) - BiarcList::nor\_SAE (function) - BiarcList::nor\_SAE\_D (function) - BiarcList::nor\_SAE\_DD (function) - BiarcList::nor\_SAE\_DDD (function) - BiarcList::numSegments (function), [1] - BiarcList::nx\_Begin\_ISO (function) - BiarcList::nx\_Begin\_SAE (function) - BiarcList::nx\_End\_ISO (function) - BiarcList::nx\_End\_SAE (function) - BiarcList::nx\_ISO (function) - BiarcList::nx\_ISO\_D (function) - BiarcList::nx\_ISO\_DD (function) - BiarcList::nx\_ISO\_DDD (function) - BiarcList::nx\_SAE (function) - BiarcList::nx\_SAE\_D (function) - BiarcList::nx\_SAE\_DD (function) - BiarcList::nx\_SAE\_DDD (function) - BiarcList::ny\_Begin\_ISO (function) - BiarcList::ny\_Begin\_SAE (function) - BiarcList::ny\_End\_ISO (function) - BiarcList::ny\_End\_SAE (function) - BiarcList::ny\_ISO (function) - BiarcList::ny\_ISO\_D (function) - BiarcList::ny\_ISO\_DD (function) - BiarcList::ny\_ISO\_DDD (function) - BiarcList::ny\_SAE (function) - BiarcList::ny\_SAE\_D (function) - BiarcList::ny\_SAE\_DD (function) - BiarcList::ny\_SAE\_DDD (function) - BiarcList::obj\_handle (function) - BiarcList::operator<< (function) - BiarcList::operator= (function) - BiarcList::plot (function) - BiarcList::plot\_offs (function) - BiarcList::plotAngle (function) - BiarcList::plotBBox (function) - BiarcList::plotCurvature (function) - BiarcList::plotNormal (function) - BiarcList::plotTBox (function) - BiarcList::plotTriangles (function) - BiarcList::points (function) - BiarcList::push\_back (function), [1], [2], [3], [4] - BiarcList::push\_back\_G1 (function), [1] - BiarcList::reserve (function), [1] - BiarcList::reverse (function), [1] - BiarcList::rotate (function), [1] - BiarcList::save (function) - BiarcList::scale (function), [1] - BiarcList::segment\_length (function) - BiarcList::segment\_length\_ISO (function) - BiarcList::tg (function) - BiarcList::tg\_D (function) - BiarcList::tg\_DD (function) - BiarcList::tg\_DDD (function) - BiarcList::theta (function), [1] - BiarcList::theta\_D (function), [1] - BiarcList::theta\_DD (function), [1] - BiarcList::theta\_DDD (function), [1] - BiarcList::thetaBegin (function), [1] - BiarcList::thetaEnd (function), [1] - BiarcList::translate (function), [1] - BiarcList::trim (function), [1] - BiarcList::tx (function) - BiarcList::tx\_Begin (function) - BiarcList::tx\_D (function) - BiarcList::tx\_DD (function) - BiarcList::tx\_DDD (function) - BiarcList::tx\_End (function) - BiarcList::ty (function) - BiarcList::ty\_Begin (function) - BiarcList::ty\_D (function) - BiarcList::ty\_DD (function) - BiarcList::ty\_DDD (function) - BiarcList::ty\_End (function) - BiarcList::type (function) - BiarcList::X (function) - BiarcList::X\_D (function) - BiarcList::X\_DD (function) - BiarcList::X\_DDD (function) - BiarcList::X\_ISO (function) - BiarcList::X\_ISO\_D (function) - BiarcList::X\_ISO\_DD (function) - BiarcList::X\_ISO\_DDD (function) - BiarcList::X\_SAE (function) - BiarcList::X\_SAE\_D (function) - BiarcList::X\_SAE\_DD (function) - BiarcList::X\_SAE\_DDD (function) - BiarcList::xBegin (function), [1] - BiarcList::xBegin\_ISO (function) - BiarcList::xBegin\_SAE (function) - BiarcList::xEnd (function), [1] - BiarcList::xEnd\_ISO (function) - BiarcList::xEnd\_SAE (function) - BiarcList::xyBegin (function) - BiarcList::xyEnd (function) - BiarcList::Y (function) - BiarcList::Y\_D (function) - BiarcList::Y\_DD (function) - BiarcList::Y\_DDD (function) - BiarcList::Y\_ISO (function) - BiarcList::Y\_ISO\_D (function) - BiarcList::Y\_ISO\_DD (function) - BiarcList::Y\_ISO\_DDD (function) - BiarcList::Y\_SAE (function) - BiarcList::Y\_SAE\_D (function) - BiarcList::Y\_SAE\_DD (function) - BiarcList::Y\_SAE\_DDD (function) - BiarcList::yBegin (function), [1] - BiarcList::yBegin\_ISO (function) - BiarcList::yBegin\_SAE (function) - BiarcList::yEnd (function), [1] - BiarcList::yEnd\_ISO (function) - BiarcList::yEnd\_SAE (function) - BiarcList::yesAABBtree (function) - BiarcList::~BiarcList (function) - build\_guess\_theta (function) |

## C

|  |  |
| --- | --- |
| - CircleArc (class), [1] - CircleArc::bbox (function), [1] - CircleArc::bbox\_ISO (function) - CircleArc::bbox\_SAE (function) - CircleArc::bbTriangle (function), [1], [2] - CircleArc::bbTriangle\_ISO (function), [1], [2] - CircleArc::bbTriangle\_SAE (function), [1], [2] - CircleArc::bbTriangles (function), [1] - CircleArc::bbTriangles\_ISO (function) - CircleArc::bbTriangles\_SAE (function) - CircleArc::build (function), [1] - CircleArc::build\_3P (function), [1] - CircleArc::build\_G1 (function), [1] - CircleArc::center (function) - CircleArc::changeCurvilinearOrigin (function), [1] - CircleArc::changeOrigin (function), [1] - CircleArc::CircleArc (function), [1], [2], [3], [4], [5] - CircleArc::closestPoint (function) - CircleArc::closestPoint\_ISO (function), [1] - CircleArc::closestPoint\_SAE (function), [1] - CircleArc::collision (function), [1], [2] - CircleArc::collision\_ISO (function), [1] - CircleArc::collision\_SAE (function) - CircleArc::copy (function), [1] - CircleArc::cosTheta0 (function) - CircleArc::curvature (function) - CircleArc::delta\_theta (function) - CircleArc::deltaTheta (function) - CircleArc::distance (function), [1] - CircleArc::distance\_ISO (function) - CircleArc::distance\_SAE (function) - CircleArc::eval (function), [1] - CircleArc::eval\_D (function), [1] - CircleArc::eval\_DD (function), [1] - CircleArc::eval\_DDD (function), [1] - CircleArc::eval\_ISO (function) - CircleArc::eval\_ISO\_D (function) - CircleArc::eval\_ISO\_DD (function) - CircleArc::eval\_ISO\_DDD (function) - CircleArc::eval\_SAE (function) - CircleArc::eval\_SAE\_D (function) - CircleArc::eval\_SAE\_DD (function) - CircleArc::eval\_SAE\_DDD (function) - CircleArc::evaluate (function), [1] - CircleArc::evaluate\_ISO (function) - CircleArc::evaluate\_SAE (function) - CircleArc::find\_coord (function) - CircleArc::findST\_ISO (function) - CircleArc::findST\_SAE (function) - CircleArc::info (function), [1] - CircleArc::intersect (function), [1], [2] - CircleArc::intersect\_ISO (function), [1] - CircleArc::intersect\_SAE (function) - CircleArc::is\_type (function) - CircleArc::kappa (function), [1] - CircleArc::kappa\_D (function), [1] - CircleArc::kappa\_DD (function), [1] - CircleArc::kappaBegin (function), [1] - CircleArc::kappaEnd (function), [1] - CircleArc::length (function), [1] - CircleArc::length\_ISO (function) - CircleArc::length\_SAE (function) - CircleArc::lenTolerance (function) - CircleArc::noAABBtree (function) - CircleArc::nor\_ISO (function) - CircleArc::nor\_ISO\_D (function) - CircleArc::nor\_ISO\_DD (function) - CircleArc::nor\_ISO\_DDD (function) - CircleArc::nor\_SAE (function) - CircleArc::nor\_SAE\_D (function) - CircleArc::nor\_SAE\_DD (function) - CircleArc::nor\_SAE\_DDD (function) - CircleArc::nx\_Begin\_ISO (function) - CircleArc::nx\_Begin\_SAE (function) - CircleArc::nx\_End\_ISO (function) - CircleArc::nx\_End\_SAE (function) - CircleArc::nx\_ISO (function) - CircleArc::nx\_ISO\_D (function) - CircleArc::nx\_ISO\_DD (function) - CircleArc::nx\_ISO\_DDD (function) - CircleArc::nx\_SAE (function) - CircleArc::nx\_SAE\_D (function) - CircleArc::nx\_SAE\_DD (function) - CircleArc::nx\_SAE\_DDD (function) - CircleArc::ny\_Begin\_ISO (function) - CircleArc::ny\_Begin\_SAE (function) - CircleArc::ny\_End\_ISO (function) - CircleArc::ny\_End\_SAE (function) - CircleArc::ny\_ISO (function) - CircleArc::ny\_ISO\_D (function) - CircleArc::ny\_ISO\_DD (function) - CircleArc::ny\_ISO\_DDD (function) - CircleArc::ny\_SAE (function) - CircleArc::ny\_SAE\_D (function) - CircleArc::ny\_SAE\_DD (function) - CircleArc::ny\_SAE\_DDD (function) - CircleArc::obj\_handle (function) - CircleArc::operator<< (function) - CircleArc::operator= (function) - CircleArc::paramNURBS (function) - CircleArc::plot (function) - CircleArc::plotBBox (function) - CircleArc::plotPolygon (function) - CircleArc::plotTBox (function) - CircleArc::plotTriangles (function) - CircleArc::points (function) - CircleArc::ray (function) - CircleArc::reverse (function), [1] - CircleArc::rotate (function), [1] - CircleArc::scale (function), [1] - CircleArc::sinTheta0 (function) - CircleArc::tg (function) - CircleArc::tg\_D (function) - CircleArc::tg\_DD (function) - CircleArc::tg\_DDD (function) - CircleArc::theta (function), [1] - CircleArc::theta\_D (function), [1] - CircleArc::theta\_DD (function), [1] - CircleArc::theta\_DDD (function), [1] - CircleArc::thetaBegin (function), [1] - CircleArc::thetaEnd (function), [1] - CircleArc::thetaMinMax (function) - CircleArc::thetaTotalVariation (function) - CircleArc::to\_nurbs (function) - CircleArc::toNURBS (function) - CircleArc::translate (function), [1] - CircleArc::trim (function), [1] - CircleArc::tx (function) - CircleArc::tx\_Begin (function) - CircleArc::tx\_D (function) - CircleArc::tx\_DD (function) - CircleArc::tx\_DDD (function) - CircleArc::tx\_End (function) - CircleArc::ty (function) - CircleArc::ty\_Begin (function) - CircleArc::ty\_D (function) - CircleArc::ty\_DD (function) - CircleArc::ty\_DDD (function) - CircleArc::ty\_End (function) - CircleArc::type (function) - CircleArc::X (function) - CircleArc::X\_D (function) - CircleArc::X\_DD (function) - CircleArc::X\_DDD (function) - CircleArc::X\_ISO (function) - CircleArc::X\_ISO\_D (function) - CircleArc::X\_ISO\_DD (function) - CircleArc::X\_ISO\_DDD (function) - CircleArc::X\_SAE (function) - CircleArc::X\_SAE\_D (function) - CircleArc::X\_SAE\_DD (function) - CircleArc::X\_SAE\_DDD (function) - CircleArc::xBegin (function), [1] - CircleArc::xBegin\_ISO (function) - CircleArc::xBegin\_SAE (function) - CircleArc::xEnd (function), [1] - CircleArc::xEnd\_ISO (function) - CircleArc::xEnd\_SAE (function) - CircleArc::xyBegin (function) - CircleArc::xyEnd (function) - CircleArc::Y (function) - CircleArc::Y\_D (function) - CircleArc::Y\_DD (function) - CircleArc::Y\_DDD (function) - CircleArc::Y\_ISO (function) - CircleArc::Y\_ISO\_D (function) - CircleArc::Y\_ISO\_DD (function) - CircleArc::Y\_ISO\_DDD (function) - CircleArc::Y\_SAE (function) - CircleArc::Y\_SAE\_D (function) - CircleArc::Y\_SAE\_DD (function) - CircleArc::Y\_SAE\_DDD (function) - CircleArc::yBegin (function), [1] - CircleArc::yBegin\_ISO (function) - CircleArc::yBegin\_SAE (function) - CircleArc::yEnd (function), [1] - CircleArc::yEnd\_ISO (function) - CircleArc::yEnd\_SAE (function) - CircleArc::yesAABBtree (function) - ClothoidCurve (class), [1] - ClothoidCurve::approximate\_collision\_ISO (function) - ClothoidCurve::bbox (function), [1] - ClothoidCurve::bbox\_ISO (function) - ClothoidCurve::bbox\_SAE (function) - ClothoidCurve::bbTriangle (function), [1] - ClothoidCurve::bbTriangle\_ISO (function), [1] - ClothoidCurve::bbTriangle\_SAE (function), [1] - ClothoidCurve::bbTriangles (function), [1] - ClothoidCurve::bbTriangles\_ISO (function) - ClothoidCurve::bbTriangles\_SAE (function) - ClothoidCurve::build (function), [1], [2], [3] - ClothoidCurve::build\_forward (function), [1] - ClothoidCurve::build\_G1 (function), [1] - ClothoidCurve::build\_G1\_D (function) - ClothoidCurve::changeCurvilinearOrigin (function), [1] - ClothoidCurve::changeOrigin (function), [1] - ClothoidCurve::closestPoint (function), [1] - ClothoidCurve::closestPoint\_ISO (function), [1] - ClothoidCurve::closestPoint\_SAE (function), [1] - ClothoidCurve::closestPointBySample (function), [1] - ClothoidCurve::ClothoidCurve (function), [1], [2], [3], [4], [5], [6], [7] - ClothoidCurve::collision (function), [1], [2] - ClothoidCurve::collision\_ISO (function), [1] - ClothoidCurve::collision\_SAE (function) - ClothoidCurve::copy (function), [1] - ClothoidCurve::curvatureMinMax (function) - ClothoidCurve::curvatureTotalVariation (function) - ClothoidCurve::deltaTheta (function) - ClothoidCurve::distance (function), [1] - ClothoidCurve::distance\_ISO (function) - ClothoidCurve::distance\_SAE (function) - ClothoidCurve::distanceBySample (function), [1], [2] - ClothoidCurve::dkappa (function), [1] - ClothoidCurve::eval (function), [1] - ClothoidCurve::eval\_D (function), [1] - ClothoidCurve::eval\_DD (function), [1] - ClothoidCurve::eval\_DDD (function), [1] - ClothoidCurve::eval\_ISO (function) - ClothoidCurve::eval\_ISO\_D (function) - ClothoidCurve::eval\_ISO\_DD (function) - ClothoidCurve::eval\_ISO\_DDD (function) - ClothoidCurve::eval\_SAE (function) - ClothoidCurve::eval\_SAE\_D (function) - ClothoidCurve::eval\_SAE\_DD (function) - ClothoidCurve::eval\_SAE\_DDD (function) - ClothoidCurve::evaluate (function), [1] - ClothoidCurve::evaluate\_ISO (function) - ClothoidCurve::evaluate\_SAE (function) - ClothoidCurve::find\_coord (function) - ClothoidCurve::findST\_ISO (function) - ClothoidCurve::findST\_SAE (function) - ClothoidCurve::getPars (function) - ClothoidCurve::infinity (function) - ClothoidCurve::info (function), [1] - ClothoidCurve::integralCurvature2 (function) - ClothoidCurve::integralJerk2 (function) - ClothoidCurve::integralSnap2 (function) - ClothoidCurve::intersect (function), [1], [2] - ClothoidCurve::intersect\_ISO (function), [1] - ClothoidCurve::intersect\_SAE (function) - ClothoidCurve::is\_type (function) - ClothoidCurve::kappa (function), [1] - ClothoidCurve::kappa\_D (function), [1] - ClothoidCurve::kappa\_DD (function), [1] - ClothoidCurve::kappaBegin (function), [1] - ClothoidCurve::kappaEnd (function), [1] - ClothoidCurve::length (function), [1] - ClothoidCurve::length\_ISO (function) - ClothoidCurve::length\_SAE (function) - ClothoidCurve::noAABBtree (function) - ClothoidCurve::nor\_ISO (function) - ClothoidCurve::nor\_ISO\_D (function) - ClothoidCurve::nor\_ISO\_DD (function) - ClothoidCurve::nor\_ISO\_DDD (function) - ClothoidCurve::nor\_SAE (function) - ClothoidCurve::nor\_SAE\_D (function) - ClothoidCurve::nor\_SAE\_DD (function) - ClothoidCurve::nor\_SAE\_DDD (function) - ClothoidCurve::nx\_Begin\_ISO (function) - ClothoidCurve::nx\_Begin\_SAE (function) - ClothoidCurve::nx\_End\_ISO (function) - ClothoidCurve::nx\_End\_SAE (function) - ClothoidCurve::nx\_ISO (function) - ClothoidCurve::nx\_ISO\_D (function) - ClothoidCurve::nx\_ISO\_DD (function) - ClothoidCurve::nx\_ISO\_DDD (function) - ClothoidCurve::nx\_SAE (function) - ClothoidCurve::nx\_SAE\_D (function) - ClothoidCurve::nx\_SAE\_DD (function) - ClothoidCurve::nx\_SAE\_DDD (function) - ClothoidCurve::ny\_Begin\_ISO (function) - ClothoidCurve::ny\_Begin\_SAE (function) - ClothoidCurve::ny\_End\_ISO (function) - ClothoidCurve::ny\_End\_SAE (function) - ClothoidCurve::ny\_ISO (function) - ClothoidCurve::ny\_ISO\_D (function) - ClothoidCurve::ny\_ISO\_DD (function) - ClothoidCurve::ny\_ISO\_DDD (function) - ClothoidCurve::ny\_SAE (function) - ClothoidCurve::ny\_SAE\_D (function) - ClothoidCurve::ny\_SAE\_DD (function) - ClothoidCurve::ny\_SAE\_DDD (function) - ClothoidCurve::obj\_handle (function) - ClothoidCurve::operator<< (function) - ClothoidCurve::operator= (function) - ClothoidCurve::optimized\_sample (function) - ClothoidCurve::optimized\_sample\_ISO (function) - ClothoidCurve::optimized\_sample\_SAE (function) - ClothoidCurve::Pinfinity (function) - ClothoidCurve::plot (function) - ClothoidCurve::plot\_offs (function) - ClothoidCurve::plotAngle (function) - ClothoidCurve::plotBBox (function) - ClothoidCurve::plotCurvature (function) - ClothoidCurve::plotNormal (function) - ClothoidCurve::plotTBox (function) - ClothoidCurve::plotTriangles (function) - ClothoidCurve::points (function) - ClothoidCurve::reverse (function), [1] - ClothoidCurve::rotate (function), [1] - ClothoidCurve::scale (function), [1] - ClothoidCurve::tg (function) - ClothoidCurve::tg\_D (function) - ClothoidCurve::tg\_DD (function) - ClothoidCurve::tg\_DDD (function) - ClothoidCurve::theta (function), [1] - ClothoidCurve::theta\_D (function), [1] - ClothoidCurve::theta\_DD (function), [1] - ClothoidCurve::theta\_DDD (function), [1] - ClothoidCurve::thetaBegin (function), [1] - ClothoidCurve::thetaEnd (function), [1] - ClothoidCurve::thetaMinMax (function) - ClothoidCurve::thetaTotalVariation (function) - ClothoidCurve::translate (function), [1] - ClothoidCurve::trim (function), [1] - ClothoidCurve::tx (function) - ClothoidCurve::tx\_Begin (function) - ClothoidCurve::tx\_D (function) - ClothoidCurve::tx\_DD (function) - ClothoidCurve::tx\_DDD (function) - ClothoidCurve::tx\_End (function) - ClothoidCurve::ty (function) - ClothoidCurve::ty\_Begin (function) - ClothoidCurve::ty\_D (function) - ClothoidCurve::ty\_DD (function) - ClothoidCurve::ty\_DDD (function) - ClothoidCurve::ty\_End (function) - ClothoidCurve::type (function) - ClothoidCurve::X (function) - ClothoidCurve::X\_D (function) - ClothoidCurve::X\_DD (function) - ClothoidCurve::X\_DDD (function) - ClothoidCurve::X\_ISO (function) - ClothoidCurve::X\_ISO\_D (function) - ClothoidCurve::X\_ISO\_DD (function) - ClothoidCurve::X\_ISO\_DDD (function) - ClothoidCurve::X\_SAE (function) - ClothoidCurve::X\_SAE\_D (function) - ClothoidCurve::X\_SAE\_DD (function) - ClothoidCurve::X\_SAE\_DDD (function) - ClothoidCurve::xBegin (function), [1] - ClothoidCurve::xBegin\_ISO (function) - ClothoidCurve::xBegin\_SAE (function) - ClothoidCurve::xEnd (function), [1] - ClothoidCurve::xEnd\_ISO (function) - ClothoidCurve::xEnd\_SAE (function) - ClothoidCurve::xyBegin (function) - ClothoidCurve::xyEnd (function) - ClothoidCurve::Y (function) - ClothoidCurve::Y\_D (function) - ClothoidCurve::Y\_DD (function) | - ClothoidCurve::Y\_DDD (function) - ClothoidCurve::Y\_ISO (function) - ClothoidCurve::Y\_ISO\_D (function) - ClothoidCurve::Y\_ISO\_DD (function) - ClothoidCurve::Y\_ISO\_DDD (function) - ClothoidCurve::Y\_SAE (function) - ClothoidCurve::Y\_SAE\_D (function) - ClothoidCurve::Y\_SAE\_DD (function) - ClothoidCurve::Y\_SAE\_DDD (function) - ClothoidCurve::yBegin (function), [1] - ClothoidCurve::yBegin\_ISO (function) - ClothoidCurve::yBegin\_SAE (function) - ClothoidCurve::yEnd (function), [1] - ClothoidCurve::yEnd\_ISO (function) - ClothoidCurve::yEnd\_SAE (function) - ClothoidCurve::yesAABBtree (function) - ClothoidList (class), [1] - ClothoidList::append (function) - ClothoidList::bbox (function), [1] - ClothoidList::bbox\_ISO (function) - ClothoidList::bbox\_SAE (function) - ClothoidList::bbTriangles (function), [1] - ClothoidList::bbTriangles\_ISO (function) - ClothoidList::bbTriangles\_SAE (function) - ClothoidList::build (function), [1], [2] - ClothoidList::build\_2arcG2 (function) - ClothoidList::build\_3arcG2 (function) - ClothoidList::build\_3arcG2fixed (function) - ClothoidList::build\_CLC (function) - ClothoidList::build\_G1 (function), [1], [2] - ClothoidList::build\_raw (function), [1], [2] - ClothoidList::build\_theta (function) - ClothoidList::changeOrigin (function), [1] - ClothoidList::closestPoint (function) - ClothoidList::closestPoint\_ISO (function), [1] - ClothoidList::closestPoint\_SAE (function), [1] - ClothoidList::closestPointBySample (function) - ClothoidList::closestPointInRange (function) - ClothoidList::closestPointInRange\_ISO (function) - ClothoidList::closestPointInRange\_SAE (function) - ClothoidList::closestPointInSRange (function) - ClothoidList::closestPointInSRange\_ISO (function) - ClothoidList::closestPointInSRange\_SAE (function) - ClothoidList::closestSegment (function), [1] - ClothoidList::closure\_check (function) - ClothoidList::closure\_gap\_tx (function) - ClothoidList::closure\_gap\_ty (function) - ClothoidList::closure\_gap\_x (function) - ClothoidList::closure\_gap\_y (function) - ClothoidList::ClothoidList (function), [1], [2], [3], [4], [5], [6], [7], [8], [9] - ClothoidList::collision (function), [1], [2] - ClothoidList::collision\_ISO (function), [1] - ClothoidList::collision\_SAE (function) - ClothoidList::copy (function), [1] - ClothoidList::deltaKappa (function) - ClothoidList::deltaTheta (function) - ClothoidList::distance (function), [1] - ClothoidList::distance\_ISO (function) - ClothoidList::distance\_SAE (function) - ClothoidList::distanceBySample (function) - ClothoidList::eval (function), [1] - ClothoidList::eval\_D (function), [1] - ClothoidList::eval\_DD (function), [1] - ClothoidList::eval\_DDD (function), [1] - ClothoidList::eval\_ISO (function) - ClothoidList::eval\_ISO\_D (function) - ClothoidList::eval\_ISO\_DD (function) - ClothoidList::eval\_ISO\_DDD (function) - ClothoidList::eval\_SAE (function) - ClothoidList::eval\_SAE\_D (function) - ClothoidList::eval\_SAE\_DD (function) - ClothoidList::eval\_SAE\_DDD (function) - ClothoidList::evaluate (function), [1] - ClothoidList::evaluate\_ISO (function) - ClothoidList::evaluate\_SAE (function) - ClothoidList::export\_ruby (function), [1] - ClothoidList::export\_table (function), [1] - ClothoidList::find\_coord (function) - ClothoidList::find\_coord1 (function) - ClothoidList::findAtS (function) - ClothoidList::findST1 (function), [1] - ClothoidList::findST\_ISO (function) - ClothoidList::findST\_SAE (function) - ClothoidList::get (function), [1] - ClothoidList::getAtS (function) - ClothoidList::getDeltaKappa (function) - ClothoidList::getDeltaTheta (function) - ClothoidList::getSK (function), [1] - ClothoidList::getSTK (function), [1], [2] - ClothoidList::getXY (function), [1] - ClothoidList::info (function), [1] - ClothoidList::init (function) - ClothoidList::intersect (function), [1], [2] - ClothoidList::intersect\_ISO (function), [1] - ClothoidList::intersect\_SAE (function) - ClothoidList::is\_closed (function), [1] - ClothoidList::is\_type (function) - ClothoidList::kappa (function), [1] - ClothoidList::kappa\_D (function), [1] - ClothoidList::kappa\_DD (function), [1] - ClothoidList::kappaBegin (function), [1] - ClothoidList::kappaEnd (function), [1] - ClothoidList::length (function), [1] - ClothoidList::length\_ISO (function) - ClothoidList::length\_SAE (function) - ClothoidList::load (function), [1] - ClothoidList::make\_closed (function), [1] - ClothoidList::make\_open (function), [1] - ClothoidList::noAABBtree (function) - ClothoidList::nor\_ISO (function) - ClothoidList::nor\_ISO\_D (function) - ClothoidList::nor\_ISO\_DD (function) - ClothoidList::nor\_ISO\_DDD (function) - ClothoidList::nor\_SAE (function) - ClothoidList::nor\_SAE\_D (function) - ClothoidList::nor\_SAE\_DD (function) - ClothoidList::nor\_SAE\_DDD (function) - ClothoidList::numSegments (function), [1] - ClothoidList::nx\_Begin\_ISO (function) - ClothoidList::nx\_Begin\_SAE (function) - ClothoidList::nx\_End\_ISO (function) - ClothoidList::nx\_End\_SAE (function) - ClothoidList::nx\_ISO (function) - ClothoidList::nx\_ISO\_D (function) - ClothoidList::nx\_ISO\_DD (function) - ClothoidList::nx\_ISO\_DDD (function) - ClothoidList::nx\_SAE (function) - ClothoidList::nx\_SAE\_D (function) - ClothoidList::nx\_SAE\_DD (function) - ClothoidList::nx\_SAE\_DDD (function) - ClothoidList::ny\_Begin\_ISO (function) - ClothoidList::ny\_Begin\_SAE (function) - ClothoidList::ny\_End\_ISO (function) - ClothoidList::ny\_End\_SAE (function) - ClothoidList::ny\_ISO (function) - ClothoidList::ny\_ISO\_D (function) - ClothoidList::ny\_ISO\_DD (function) - ClothoidList::ny\_ISO\_DDD (function) - ClothoidList::ny\_SAE (function) - ClothoidList::ny\_SAE\_D (function) - ClothoidList::ny\_SAE\_DD (function) - ClothoidList::ny\_SAE\_DDD (function) - ClothoidList::obj\_handle (function) - ClothoidList::operator<< (function) - ClothoidList::operator= (function) - ClothoidList::plot (function) - ClothoidList::plot\_offs (function) - ClothoidList::plotAngle (function) - ClothoidList::plotBBox (function) - ClothoidList::plotCurvature (function) - ClothoidList::plotNormal (function) - ClothoidList::plotTBox (function) - ClothoidList::plotTriangles (function) - ClothoidList::points (function) - ClothoidList::push\_back (function), [1], [2], [3], [4], [5], [6], [7], [8], [9] - ClothoidList::push\_back\_G1 (function), [1], [2] - ClothoidList::reserve (function), [1] - ClothoidList::reverse (function), [1] - ClothoidList::rotate (function), [1] - ClothoidList::s\_to\_index (function) - ClothoidList::save (function), [1] - ClothoidList::saveClothoids (function) - ClothoidList::saveSampled (function) - ClothoidList::scale (function), [1] - ClothoidList::segment\_length (function) - ClothoidList::segment\_length\_ISO (function) - ClothoidList::segment\_length\_SAE (function) - ClothoidList::tg (function) - ClothoidList::tg\_D (function) - ClothoidList::tg\_DD (function) - ClothoidList::tg\_DDD (function) - ClothoidList::theta (function), [1] - ClothoidList::theta\_D (function), [1] - ClothoidList::theta\_DD (function), [1] - ClothoidList::theta\_DDD (function), [1] - ClothoidList::thetaBegin (function), [1] - ClothoidList::thetaEnd (function), [1] - ClothoidList::translate (function), [1] - ClothoidList::trim (function), [1], [2] - ClothoidList::tx (function) - ClothoidList::tx\_Begin (function) - ClothoidList::tx\_D (function) - ClothoidList::tx\_DD (function) - ClothoidList::tx\_DDD (function) - ClothoidList::tx\_End (function) - ClothoidList::ty (function) - ClothoidList::ty\_Begin (function) - ClothoidList::ty\_D (function) - ClothoidList::ty\_DD (function) - ClothoidList::ty\_DDD (function) - ClothoidList::ty\_End (function) - ClothoidList::type (function) - ClothoidList::wrap\_in\_range (function) - ClothoidList::X (function) - ClothoidList::X\_D (function) - ClothoidList::X\_DD (function) - ClothoidList::X\_DDD (function) - ClothoidList::X\_ISO (function) - ClothoidList::X\_ISO\_D (function) - ClothoidList::X\_ISO\_DD (function) - ClothoidList::X\_ISO\_DDD (function) - ClothoidList::X\_SAE (function) - ClothoidList::X\_SAE\_D (function) - ClothoidList::X\_SAE\_DD (function) - ClothoidList::X\_SAE\_DDD (function) - ClothoidList::xBegin (function), [1] - ClothoidList::xBegin\_ISO (function) - ClothoidList::xBegin\_SAE (function) - ClothoidList::xEnd (function), [1] - ClothoidList::xEnd\_ISO (function) - ClothoidList::xEnd\_SAE (function) - ClothoidList::xyBegin (function) - ClothoidList::xyEnd (function) - ClothoidList::Y (function) - ClothoidList::Y\_D (function) - ClothoidList::Y\_DD (function) - ClothoidList::Y\_DDD (function) - ClothoidList::Y\_ISO (function) - ClothoidList::Y\_ISO\_D (function) - ClothoidList::Y\_ISO\_DD (function) - ClothoidList::Y\_ISO\_DDD (function) - ClothoidList::Y\_SAE (function) - ClothoidList::Y\_SAE\_D (function) - ClothoidList::Y\_SAE\_DD (function) - ClothoidList::Y\_SAE\_DDD (function) - ClothoidList::yBegin (function), [1] - ClothoidList::yBegin\_ISO (function) - ClothoidList::yBegin\_SAE (function) - ClothoidList::yEnd (function), [1] - ClothoidList::yEnd\_ISO (function) - ClothoidList::yEnd\_SAE (function) - ClothoidList::yesAABBtree (function) - ClothoidList::~ClothoidList (function) - CLOTHOIDS\_dot\_HH (C macro) - ClothoidSplineG2 (class), [1] - ClothoidSplineG2::build (function), [1] - ClothoidSplineG2::build\_internal (function) - ClothoidSplineG2::build\_internal2 (function) - ClothoidSplineG2::buildP1 (function) - ClothoidSplineG2::buildP2 (function) - ClothoidSplineG2::buildP3 (function) - ClothoidSplineG2::buildP4 (function) - ClothoidSplineG2::buildP5 (function) - ClothoidSplineG2::buildP6 (function) - ClothoidSplineG2::buildP7 (function) - ClothoidSplineG2::buildP8 (function) - ClothoidSplineG2::buildP9 (function) - ClothoidSplineG2::ClothoidSplineG2 (function), [1] - ClothoidSplineG2::con (function) - ClothoidSplineG2::constraints (function) - ClothoidSplineG2::dims (function) - ClothoidSplineG2::gradient (function) - ClothoidSplineG2::guess (function), [1] - ClothoidSplineG2::info (function) - ClothoidSplineG2::ipopt (function) - ClothoidSplineG2::ipopt\_check (function) - ClothoidSplineG2::jacobian (function) - ClothoidSplineG2::jacobian\_nnz (function) - ClothoidSplineG2::jacobian\_pattern (function) - ClothoidSplineG2::jacobian\_pattern\_matlab (function) - ClothoidSplineG2::nlsys (function) - ClothoidSplineG2::numConstraints (function) - ClothoidSplineG2::numPnts (function) - ClothoidSplineG2::numTheta (function) - ClothoidSplineG2::obj (function) - ClothoidSplineG2::objective (function) - ClothoidSplineG2::operator<< (function) - ClothoidSplineG2::setP1 (function) - ClothoidSplineG2::setP2 (function) - ClothoidSplineG2::setP3 (function) - ClothoidSplineG2::setP4 (function) - ClothoidSplineG2::setP5 (function) - ClothoidSplineG2::setP6 (function) - ClothoidSplineG2::setP7 (function) - ClothoidSplineG2::setP8 (function) - ClothoidSplineG2::setP9 (function) - ClothoidSplineG2::TargetType (C++ enum) - ClothoidSplineG2::TargetType::P1 (C++ enumerator) - ClothoidSplineG2::TargetType::P2 (C++ enumerator) - ClothoidSplineG2::TargetType::P3 (C++ enumerator) - ClothoidSplineG2::TargetType::P4 (C++ enumerator) - ClothoidSplineG2::TargetType::P5 (C++ enumerator) - ClothoidSplineG2::TargetType::P6 (C++ enumerator) - ClothoidSplineG2::TargetType::P7 (C++ enumerator) - ClothoidSplineG2::TargetType::P8 (C++ enumerator) - ClothoidSplineG2::TargetType::P9 (C++ enumerator) - ClothoidSplineG2::verbose (function) - ClothoidSplineG2::~ClothoidSplineG2 (function) - collision (function) - collision\_ISO (function) - collision\_SAE (function) - Cosc (function) - Cosc\_D (function) - Cosc\_DD (function) - Cosc\_DDD (function) - CurveBase (class) - CurveBase::bbox (function) - CurveBase::bbTriangles (function) - CurveBase::changeOrigin (function) - CurveBase::closestPoint (function) - CurveBase::collision (function) - CurveBase::copy (function) - CurveBase::CurveBase (function) - CurveBase::distance (function) - CurveBase::eval (function) - CurveBase::eval\_D (function) - CurveBase::eval\_DD (function) - CurveBase::eval\_DDD (function) - CurveBase::evaluate (function) - CurveBase::find\_coord (function) - CurveBase::info (function) - CurveBase::intersect (function) - CurveBase::kappa (function) - CurveBase::kappa\_D (function) - CurveBase::kappa\_DD (function) - CurveBase::kappaBegin (function) - CurveBase::kappaEnd (function) - CurveBase::length (function) - CurveBase::noAABBtree (function) - CurveBase::obj\_handle (function) - CurveBase::plotBBox (function) - CurveBase::plotTBox (function) - CurveBase::plotTriangles (function) - CurveBase::points (function) - CurveBase::reverse (function) - CurveBase::rotate (function) - CurveBase::scale (function) - CurveBase::theta (function) - CurveBase::theta\_D (function) - CurveBase::theta\_DD (function) - CurveBase::theta\_DDD (function) - CurveBase::thetaBegin (function) - CurveBase::thetaEnd (function) - CurveBase::translate (function) - CurveBase::trim (function) - CurveBase::xBegin (function) - CurveBase::xEnd (function) - CurveBase::xyBegin (function) - CurveBase::xyEnd (function) - CurveBase::yBegin (function) - CurveBase::yEnd (function) - CurveBase::yesAABBtree (function) - CurveType (C++ enum) - CurveType::G2LIB\_BIARC (C++ enumerator) - CurveType::G2LIB\_BIARC\_LIST (C++ enumerator) - CurveType::G2LIB\_CIRCLE (C++ enumerator) - CurveType::G2LIB\_CLOTHOID (C++ enumerator) - CurveType::G2LIB\_CLOTHOID\_LIST (C++ enumerator) - CurveType::G2LIB\_LINE (C++ enumerator) - CurveType::G2LIB\_POLYLINE (C++ enumerator) - CurveType\_name (C++ member) |

## F

- FresnelCS (function), [1]

## G

|  |  |
| --- | --- |
| - G2LIB\_DEBUG\_MESSAGE (C macro) - G2LIB\_USE\_CXX11 (C macro) - G2solve2arc (class) - G2solve2arc::build (function) - G2solve2arc::G2solve2arc (function) - G2solve2arc::getS0 (function) - G2solve2arc::getS1 (function) - G2solve2arc::setMaxIter (function) - G2solve2arc::setTolerance (function) - G2solve2arc::solve (function) - G2solve2arc::~G2solve2arc (function) - G2solve3arc (class) - G2solve3arc::build (function) - G2solve3arc::build\_fixed\_length (function) - G2solve3arc::curvatureMinMax (function) - G2solve3arc::curvatureTotalVariation (function) - G2solve3arc::deltaTheta (function) - G2solve3arc::eval (function), [1] - G2solve3arc::eval\_D (function) - G2solve3arc::eval\_DD (function) - G2solve3arc::eval\_DDD (function) - G2solve3arc::eval\_ISO (function) - G2solve3arc::eval\_ISO\_D (function) - G2solve3arc::eval\_ISO\_DD (function) - G2solve3arc::eval\_ISO\_DDD (function) - G2solve3arc::G2solve3arc (function) - G2solve3arc::getS0 (function) - G2solve3arc::getS1 (function) - G2solve3arc::getSM (function) - G2solve3arc::integralCurvature2 (function) - G2solve3arc::integralJerk2 (function) - G2solve3arc::integralSnap2 (function) - G2solve3arc::kappaBegin (function) - G2solve3arc::kappaEnd (function) - G2solve3arc::operator<< (function) | - G2solve3arc::reverse (function) - G2solve3arc::rotate (function) - G2solve3arc::save (function) - G2solve3arc::setMaxIter (function) - G2solve3arc::setTolerance (function) - G2solve3arc::theta (function) - G2solve3arc::theta\_D (function) - G2solve3arc::theta\_DD (function) - G2solve3arc::theta\_DDD (function) - G2solve3arc::thetaBegin (function) - G2solve3arc::thetaEnd (function) - G2solve3arc::thetaMinMax (function) - G2solve3arc::thetaTotalVariation (function) - G2solve3arc::totalLength (function) - G2solve3arc::translate (function) - G2solve3arc::X (function) - G2solve3arc::xBegin (function) - G2solve3arc::xEnd (function) - G2solve3arc::Y (function) - G2solve3arc::yBegin (function) - G2solve3arc::yEnd (function) - G2solve3arc::~G2solve3arc (function) - G2solveCLC (class) - G2solveCLC::build (function) - G2solveCLC::G2solveCLC (function) - G2solveCLC::getS0 (function) - G2solveCLC::getS1 (function) - G2solveCLC::getSM (function) - G2solveCLC::save (function) - G2solveCLC::setMaxIter (function) - G2solveCLC::setTolerance (function) - G2solveCLC::solve (function) - G2solveCLC::~G2solveCLC (function) - GeneralizedFresnelCS (function), [1] - GLIB2\_TOL\_ANGLE (C macro) |

## I

|  |  |
| --- | --- |
| - int\_type (C++ type) - intersect (function) - intersect\_ISO (function) - intersect\_SAE (function) - intersect\_with\_AABBtree (C++ member) | - intersectCircleCircle (function) - IntersectList (C++ type) - Ipair (C++ type) - isCounterClockwise (function) - isPointInTriangle (function) - istream\_type (C++ type) |

## L

|  |  |
| --- | --- |
| - LineSegment (class), [1] - LineSegment::bbox (function), [1] - LineSegment::bbox\_ISO (function) - LineSegment::bbox\_SAE (function) - LineSegment::bbTriangles (function), [1] - LineSegment::bbTriangles\_ISO (function) - LineSegment::bbTriangles\_SAE (function) - LineSegment::build (function), [1] - LineSegment::build\_2P (function), [1] - LineSegment::changeOrigin (function), [1] - LineSegment::closestPoint (function) - LineSegment::closestPoint\_ISO (function), [1] - LineSegment::closestPoint\_SAE (function), [1] - LineSegment::collision (function), [1], [2] - LineSegment::collision\_ISO (function), [1] - LineSegment::collision\_SAE (function) - LineSegment::copy (function), [1] - LineSegment::distance (function), [1] - LineSegment::distance\_ISO (function) - LineSegment::distance\_SAE (function) - LineSegment::eval (function), [1] - LineSegment::eval\_D (function), [1] - LineSegment::eval\_DD (function), [1] - LineSegment::eval\_DDD (function), [1] - LineSegment::eval\_ISO (function) - LineSegment::eval\_ISO\_D (function) - LineSegment::eval\_ISO\_DD (function) - LineSegment::eval\_ISO\_DDD (function) - LineSegment::eval\_SAE (function) - LineSegment::eval\_SAE\_D (function) - LineSegment::eval\_SAE\_DD (function) - LineSegment::eval\_SAE\_DDD (function) - LineSegment::evaluate (function), [1] - LineSegment::evaluate\_ISO (function) - LineSegment::evaluate\_SAE (function) - LineSegment::find\_coord (function) - LineSegment::findST\_ISO (function) - LineSegment::findST\_SAE (function) - LineSegment::info (function), [1] - LineSegment::intersect (function), [1], [2], [3] - LineSegment::intersect\_ISO (function), [1], [2] - LineSegment::intersect\_SAE (function) - LineSegment::is\_type (function) - LineSegment::kappa (function), [1] - LineSegment::kappa\_D (function), [1] - LineSegment::kappa\_DD (function), [1] - LineSegment::kappaBegin (function), [1] - LineSegment::kappaEnd (function), [1] - LineSegment::length (function), [1] - LineSegment::length\_ISO (function) - LineSegment::length\_SAE (function) - LineSegment::LineSegment (function), [1], [2], [3], [4] - LineSegment::noAABBtree (function) - LineSegment::nor\_ISO (function) - LineSegment::nor\_ISO\_D (function) - LineSegment::nor\_ISO\_DD (function) - LineSegment::nor\_ISO\_DDD (function) - LineSegment::nor\_SAE (function) - LineSegment::nor\_SAE\_D (function) - LineSegment::nor\_SAE\_DD (function) - LineSegment::nor\_SAE\_DDD (function) - LineSegment::nx\_Begin\_ISO (function) - LineSegment::nx\_Begin\_SAE (function) - LineSegment::nx\_End\_ISO (function) - LineSegment::nx\_End\_SAE (function) - LineSegment::nx\_ISO (function) - LineSegment::nx\_ISO\_D (function) - LineSegment::nx\_ISO\_DD (function) - LineSegment::nx\_ISO\_DDD (function) - LineSegment::nx\_SAE (function) - LineSegment::nx\_SAE\_D (function) - LineSegment::nx\_SAE\_DD (function) - LineSegment::nx\_SAE\_DDD (function) - LineSegment::ny\_Begin\_ISO (function) - LineSegment::ny\_Begin\_SAE (function) - LineSegment::ny\_End\_ISO (function) - LineSegment::ny\_End\_SAE (function) - LineSegment::ny\_ISO (function) - LineSegment::ny\_ISO\_D (function) - LineSegment::ny\_ISO\_DD (function) - LineSegment::ny\_ISO\_DDD (function) - LineSegment::ny\_SAE (function) | - LineSegment::ny\_SAE\_D (function) - LineSegment::ny\_SAE\_DD (function) - LineSegment::ny\_SAE\_DDD (function) - LineSegment::obj\_handle (function) - LineSegment::operator<< (function) - LineSegment::operator= (function) - LineSegment::p1p2 (function) - LineSegment::paramNURBS (function) - LineSegment::plot (function) - LineSegment::plotBBox (function) - LineSegment::plotTBox (function) - LineSegment::plotTriangles (function) - LineSegment::points (function) - LineSegment::reverse (function), [1] - LineSegment::rotate (function), [1] - LineSegment::scale (function), [1] - LineSegment::tg (function) - LineSegment::tg\_D (function) - LineSegment::tg\_DD (function) - LineSegment::tg\_DDD (function) - LineSegment::theta (function), [1] - LineSegment::theta\_D (function), [1] - LineSegment::theta\_DD (function), [1] - LineSegment::theta\_DDD (function), [1] - LineSegment::thetaBegin (function), [1] - LineSegment::thetaEnd (function), [1] - LineSegment::to\_nurbs (function) - LineSegment::toBS (function) - LineSegment::toNURBS (function) - LineSegment::translate (function), [1] - LineSegment::trim (function), [1] - LineSegment::tx (function) - LineSegment::tx\_Begin (function) - LineSegment::tx\_D (function) - LineSegment::tx\_DD (function) - LineSegment::tx\_DDD (function) - LineSegment::tx\_End (function) - LineSegment::ty (function) - LineSegment::ty\_Begin (function) - LineSegment::ty\_D (function) - LineSegment::ty\_DD (function) - LineSegment::ty\_DDD (function) - LineSegment::ty\_End (function) - LineSegment::type (function) - LineSegment::X (function) - LineSegment::X\_D (function) - LineSegment::X\_DD (function) - LineSegment::X\_DDD (function) - LineSegment::X\_ISO (function) - LineSegment::X\_ISO\_D (function) - LineSegment::X\_ISO\_DD (function) - LineSegment::X\_ISO\_DDD (function) - LineSegment::X\_SAE (function) - LineSegment::X\_SAE\_D (function) - LineSegment::X\_SAE\_DD (function) - LineSegment::X\_SAE\_DDD (function) - LineSegment::xBegin (function), [1] - LineSegment::xBegin\_ISO (function) - LineSegment::xBegin\_SAE (function) - LineSegment::xEnd (function), [1] - LineSegment::xEnd\_ISO (function) - LineSegment::xEnd\_SAE (function) - LineSegment::xyBegin (function) - LineSegment::xyEnd (function) - LineSegment::Y (function) - LineSegment::Y\_D (function) - LineSegment::Y\_DD (function) - LineSegment::Y\_DDD (function) - LineSegment::Y\_ISO (function) - LineSegment::Y\_ISO\_D (function) - LineSegment::Y\_ISO\_DD (function) - LineSegment::Y\_ISO\_DDD (function) - LineSegment::Y\_SAE (function) - LineSegment::Y\_SAE\_D (function) - LineSegment::Y\_SAE\_DD (function) - LineSegment::Y\_SAE\_DDD (function) - LineSegment::yBegin (function), [1] - LineSegment::yBegin\_ISO (function) - LineSegment::yBegin\_SAE (function) - LineSegment::yEnd (function), [1] - LineSegment::yEnd\_ISO (function) - LineSegment::yEnd\_SAE (function) - LineSegment::yesAABBtree (function) |

## M

|  |  |
| --- | --- |
| - m\_1\_sqrt\_pi (C++ member) - machepsi (C++ member) - machepsi10 (C++ member) | - machepsi100 (C++ member) - machepsi1000 (C++ member) - minmax3 (function) |

## N

- noAABBtree (function)

## O

|  |  |
| --- | --- |
| - operator<< (function), [1], [2], [3], [4], [5], [6], [7], [8], [9] | - ostream\_type (C++ type) |

## P

|  |  |
| --- | --- |
| - pointInsideCircle (function) - PolyLine (class), [1] - PolyLine::approx (function) - PolyLine::bbox (function), [1] - PolyLine::bbox\_ISO (function) - PolyLine::bbox\_SAE (function) - PolyLine::bbTriangles (function), [1] - PolyLine::bbTriangles\_ISO (function) - PolyLine::bbTriangles\_SAE (function) - PolyLine::build (function), [1], [2], [3], [4], [5], [6] - PolyLine::build\_AABBtree (function), [1] - PolyLine::changeOrigin (function), [1] - PolyLine::closestPoint (function) - PolyLine::closestPoint\_ISO (function), [1] - PolyLine::closestPoint\_SAE (function), [1] - PolyLine::collision (function), [1], [2] - PolyLine::collision\_ISO (function), [1] - PolyLine::collision\_SAE (function) - PolyLine::copy (function), [1] - PolyLine::distance (function), [1] - PolyLine::distance\_ISO (function) - PolyLine::distance\_SAE (function) - PolyLine::eval (function), [1] - PolyLine::eval\_D (function), [1] - PolyLine::eval\_DD (function), [1] - PolyLine::eval\_DDD (function), [1] - PolyLine::eval\_ISO (function) - PolyLine::eval\_ISO\_D (function) - PolyLine::eval\_ISO\_DD (function) - PolyLine::eval\_ISO\_DDD (function) - PolyLine::eval\_SAE (function) - PolyLine::eval\_SAE\_D (function) - PolyLine::eval\_SAE\_DD (function) - PolyLine::eval\_SAE\_DDD (function) - PolyLine::evaluate (function), [1] - PolyLine::evaluate\_ISO (function) - PolyLine::evaluate\_SAE (function) - PolyLine::find\_coord (function) - PolyLine::findAtS (function) - PolyLine::findST\_ISO (function) - PolyLine::findST\_SAE (function) - PolyLine::getSegment (function) - PolyLine::info (function), [1] - PolyLine::init (function), [1] - PolyLine::intersect (function), [1], [2], [3] - PolyLine::intersect\_ISO (function), [1] - PolyLine::intersect\_SAE (function) - PolyLine::is\_type (function) - PolyLine::kappa (function), [1] - PolyLine::kappa\_D (function), [1] - PolyLine::kappa\_DD (function), [1] - PolyLine::kappaBegin (function), [1] - PolyLine::kappaEnd (function), [1] - PolyLine::length (function), [1] - PolyLine::length\_ISO (function) - PolyLine::length\_SAE (function) - PolyLine::noAABBtree (function) - PolyLine::nor\_ISO (function) - PolyLine::nor\_ISO\_D (function) - PolyLine::nor\_ISO\_DD (function) - PolyLine::nor\_ISO\_DDD (function) - PolyLine::nor\_SAE (function) - PolyLine::nor\_SAE\_D (function) - PolyLine::nor\_SAE\_DD (function) - PolyLine::nor\_SAE\_DDD (function) - PolyLine::numPoints (function) - PolyLine::numSegments (function) - PolyLine::nx\_Begin\_ISO (function) - PolyLine::nx\_Begin\_SAE (function) - PolyLine::nx\_End\_ISO (function) - PolyLine::nx\_End\_SAE (function) - PolyLine::nx\_ISO (function) - PolyLine::nx\_ISO\_D (function) - PolyLine::nx\_ISO\_DD (function) - PolyLine::nx\_ISO\_DDD (function) - PolyLine::nx\_SAE (function) - PolyLine::nx\_SAE\_D (function) - PolyLine::nx\_SAE\_DD (function) - PolyLine::nx\_SAE\_DDD (function) - PolyLine::ny\_Begin\_ISO (function) - PolyLine::ny\_Begin\_SAE (function) - PolyLine::ny\_End\_ISO (function) - PolyLine::ny\_End\_SAE (function) - PolyLine::ny\_ISO (function) - PolyLine::ny\_ISO\_D (function) - PolyLine::ny\_ISO\_DD (function) | - PolyLine::ny\_ISO\_DDD (function) - PolyLine::ny\_SAE (function) - PolyLine::ny\_SAE\_D (function) - PolyLine::ny\_SAE\_DD (function) - PolyLine::ny\_SAE\_DDD (function) - PolyLine::obj\_handle (function) - PolyLine::operator<< (function) - PolyLine::operator= (function) - PolyLine::plot (function) - PolyLine::plotBBox (function) - PolyLine::plotTBox (function) - PolyLine::plotTriangles (function) - PolyLine::points (function) - PolyLine::polygon (function), [1] - PolyLine::PolyLine (function), [1], [2], [3], [4], [5], [6], [7], [8] - PolyLine::push\_back (function), [1], [2], [3], [4], [5] - PolyLine::reverse (function), [1] - PolyLine::rotate (function), [1] - PolyLine::s\_to\_index (function) - PolyLine::scale (function), [1] - PolyLine::tg (function) - PolyLine::tg\_D (function) - PolyLine::tg\_DD (function) - PolyLine::tg\_DDD (function) - PolyLine::theta (function), [1] - PolyLine::theta\_D (function), [1] - PolyLine::theta\_DD (function), [1] - PolyLine::theta\_DDD (function), [1] - PolyLine::thetaBegin (function), [1] - PolyLine::thetaEnd (function), [1] - PolyLine::translate (function), [1] - PolyLine::trim (function), [1], [2] - PolyLine::tx (function) - PolyLine::tx\_Begin (function) - PolyLine::tx\_D (function) - PolyLine::tx\_DD (function) - PolyLine::tx\_DDD (function) - PolyLine::tx\_End (function) - PolyLine::ty (function) - PolyLine::ty\_Begin (function) - PolyLine::ty\_D (function) - PolyLine::ty\_DD (function) - PolyLine::ty\_DDD (function) - PolyLine::ty\_End (function) - PolyLine::type (function) - PolyLine::X (function) - PolyLine::X\_D (function) - PolyLine::X\_DD (function) - PolyLine::X\_DDD (function) - PolyLine::X\_ISO (function) - PolyLine::X\_ISO\_D (function) - PolyLine::X\_ISO\_DD (function) - PolyLine::X\_ISO\_DDD (function) - PolyLine::X\_SAE (function) - PolyLine::X\_SAE\_D (function) - PolyLine::X\_SAE\_DD (function) - PolyLine::X\_SAE\_DDD (function) - PolyLine::xBegin (function), [1] - PolyLine::xBegin\_ISO (function) - PolyLine::xBegin\_SAE (function) - PolyLine::xEnd (function), [1] - PolyLine::xEnd\_ISO (function) - PolyLine::xEnd\_SAE (function) - PolyLine::xyBegin (function) - PolyLine::xyEnd (function) - PolyLine::Y (function) - PolyLine::Y\_D (function) - PolyLine::Y\_DD (function) - PolyLine::Y\_DDD (function) - PolyLine::Y\_ISO (function) - PolyLine::Y\_ISO\_D (function) - PolyLine::Y\_ISO\_DD (function) - PolyLine::Y\_ISO\_DDD (function) - PolyLine::Y\_SAE (function) - PolyLine::Y\_SAE\_D (function) - PolyLine::Y\_SAE\_DD (function) - PolyLine::Y\_SAE\_DDD (function) - PolyLine::yBegin (function), [1] - PolyLine::yBegin\_ISO (function) - PolyLine::yBegin\_SAE (function) - PolyLine::yEnd (function), [1] - PolyLine::yEnd\_ISO (function) - PolyLine::yEnd\_SAE (function) - PolyLine::yesAABBtree (function) - projectPointOnCircle (function) - projectPointOnCircleArc (function) |

## R

|  |  |
| --- | --- |
| - rangeSymm (function) | - real\_type (C++ type) |

## S

|  |  |
| --- | --- |
| - Sinc (function) - Sinc\_D (function) - Sinc\_DD (function) - Sinc\_DDD (function) - Solve2x2 (class) | - Solve2x2::factorize (function) - Solve2x2::solve (function) - Solve2x2::Solve2x2 (function) - solveLinearQuadratic (function) - solveLinearQuadratic2 (function) - sqrtMachepsi (C++ member) |

## T

|  |  |
| --- | --- |
| - Triangle2D (class), [1] - Triangle2D::baricenterX (function) - Triangle2D::baricenterY (function) - Triangle2D::bbox (function) - Triangle2D::build (function), [1], [2] - Triangle2D::distanceMax (function) - Triangle2D::distanceMin (function) - Triangle2D::distMax (function) - Triangle2D::distMin (function) - Triangle2D::Icurve (function) - Triangle2D::info (function), [1] - Triangle2D::is\_type (function) - Triangle2D::isCounterClockwise (function) - Triangle2D::isInside (function), [1], [2] - Triangle2D::obj\_handle (function) - Triangle2D::operator<< (function) - Triangle2D::operator= (function) - Triangle2D::overlap (function), [1] - Triangle2D::P1 (function) | - Triangle2D::P2 (function) - Triangle2D::P3 (function) - Triangle2D::plot (function) - Triangle2D::plot2 (function) - Triangle2D::points (function) - Triangle2D::rotate (function), [1] - Triangle2D::S0 (function) - Triangle2D::S1 (function) - Triangle2D::scale (function), [1] - Triangle2D::translate (function), [1] - Triangle2D::Triangle2D (function), [1], [2], [3], [4] - Triangle2D::x1 (function) - Triangle2D::x2 (function) - Triangle2D::x3 (function) - Triangle2D::y1 (function) - Triangle2D::y2 (function) - Triangle2D::y3 (function) - Triangle2D::~Triangle2D (function) - TriTriOverlap (function) |

## X

- xy\_to\_guess\_angle (function)

## Y

- yesAABBtree (function)

### Quick search

### Table of Contents

- Matlab Interface Manual
- C++ API
- MATLAB API

«
hide menu

menu
sidebar
»

### Navigation

- index
- toc
- Clothoids »
- Index

© Copyright 2021, Enrico Bertolazzi and Marco Frego.
Created using Sphinx 4.2.0.
